# Custom G4 Microarrays Reveal Selective G-Quadruplex Recognition of Small Molecule BMVC: A Large-Scale Assessment of Ligand Binding Selectivity

**DOI:** 10.3390/molecules25153465

**Published:** 2020-07-30

**Authors:** Guanhui Wu, Desiree Tillo, Sreejana Ray, Ta-Chau Chang, John S. Schneekloth, Charles Vinson, Danzhou Yang

**Affiliations:** 1 Department of Medicinal Chemistry and Molecular Pharmacology, College of Pharmacy, Purdue University, 575 W Stadium Ave, West Lafayette, IN 47907, USA; wu1109@purdue.edu; 2 Laboratory of Metabolism, National Cancer Institute, National Institutes of Health, Bethesda, MD 20892, USA; desiree.tillo@nih.gov (D.T.); sreejana.ray@gmail.com (S.R.); vinsonc@mail.nih.gov (C.V.); 3 Institute of Atomic and Molecular Sciences, Academia Sinica, P.O. Box 23-166, Taipei 106, Taiwan; tcchang@pub.iams.sinica.edu.tw; 4 Chemical Biology Laboratory, National Cancer Institute-Frederick, Frederick, MD 21702, USA; schneeklothjs@mail.nih.gov; 5 Purdue Center for Cancer Research, West Lafayette, IN 47906, USA; 6 Department of Chemistry, Purdue University, West Lafayette, IN 47907, USA

**Keywords:** G-quadruplex, G4, microarray, ligand selectivity, BMVC, MYC

## Abstract

G-quadruplexes (G4) are considered new drug targets for human diseases such as cancer. More than 10,000 G4s have been discovered in human chromatin, posing challenges for assessing the selectivity of a G4-interactive ligand. 3,6-bis(1-Methyl-4-vinylpyridinium) carbazole diiodide (BMVC) is the first fluorescent small molecule for G4 detection in vivo. Our previous structural study shows that BMVC binds to the MYC promoter G4 (MycG4) with high specificity. Here, we utilize high-throughput, large-scale custom DNA G4 microarrays to analyze the G4-binding selectivity of BMVC. BMVC preferentially binds to the parallel MycG4 and selectively recognizes flanking sequences of parallel G4s, especially the 3′-flanking thymine. Importantly, the microarray results are confirmed by orthogonal NMR and fluorescence binding analyses. Our study demonstrates the potential of custom G4 microarrays as a platform to broadly and unbiasedly assess the binding selectivity of G4-interactive ligands, and to help understand the properties that govern molecular recognition.

## 1. Introduction

G-quadruplexes (G4s) are four-stranded secondary structures formed in guanine-rich nucleic acids [1]. The building block of G4s is the G-tetrad, consisting of four guanines connected through Hoogsteen hydrogen bonds in a cyclic coplanar arrangement [2]. A G4 structure is formed when two or more G-tetrad planes stack on top of each other and is stabilized by physiological relevant monovalent cations, especially K^+^ [3,4,5]. The biologically relevant intramolecular G4s are globular nucleic acid structures with unique folding and capping structures that provide an opportunity for selective targeting by small molecules [6,7,8].

G4 structures are involved in many cellular processes of DNA, including gene transcription [9,10], DNA replication [11], and genome stability [12,13]. In the human genome, G4 structures are prevalent in the regulatory regions and enriched in the promoters of cancer-related genes [14,15]. In particular, MYC, one of the most deregulated oncogenes in human cancer, has a DNA-G4 forming motif (MycG4) in its promoter [9,16,17,18,19,20]. Compounds that bind and stabilize the MycG4 structure have been shown to repress MYC expression and lead to cancer cell death [8,9,16]. Therefore, the MycG4 is considered an attractive target for anticancer drugs. However, over 10,000 G4 structures have been discovered in human chromatin of precancerous cells [15,21]. It is thus important to determine the selectivity of a G4-targeting compound.

**Figure 1:**
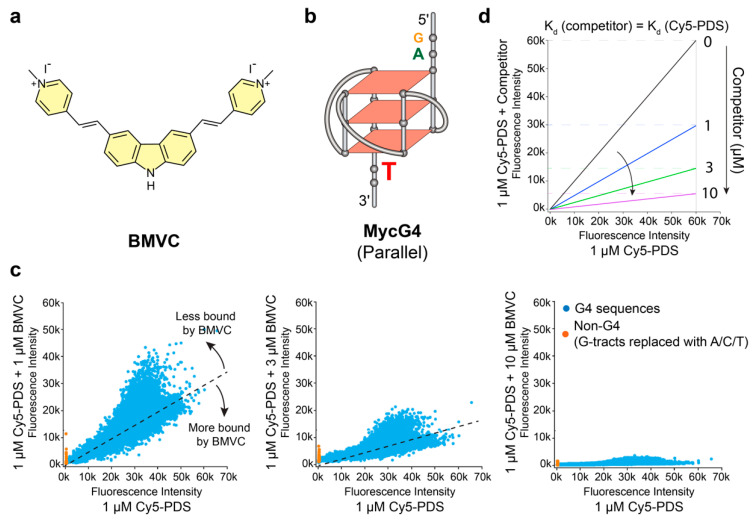
The binding of 3,6-bis(1-Methyl-4-vinylpyridinium) carbazole diiodide (BMVC) to various G4 structures differs from pyridostatin (PDS). (**a**) Chemical structure of BMVC. (**b**) MycG4, the major G-quadruplex formed in the MYC promoter NHE III_1_ in K^+^ solution, a parallel-stranded G4 structure. (**c**) Competition experiments of DNA microarrays with thousands of G4 sequences showing the differential binding of BMVC to various G4s as compared to Cy5-fluorophore (λ_ex,max_ = 647, λ_em,max_ = 665) labeled small molecule pyridostatin (Cy5-PDS), as shown by Cy5-PDS fluorescence intensity. The competition experiments were performed in the presence of 1, 3, and 10 µM BMVC. The black dashed lines represent predicted linear relationships when the binding affinities of BMVC and PDS are the same. G4-containing sequences are shown in blue spots. Non-G4 forming sequences are shown in orange spots and serve as negative controls. Each spot represents the average of two independent measurements (Figure S1). (**d**) Schematic diagram showing the predicted linear relationships when the competitor has the same binding affinities as Cy5-PDS. The competition effects can be revealed by a dose-dependent slope reduction.

3,6-bis(1-Methyl-4-vinylpyridinium) carbazole diiodide (BMVC, Figure 1a) is a G4-interactive compound and the first fluorescent probe (λ_ex,max_ = 435, λ_em,max_ = 580) to detect G4 structures in human cells [22,23,24]. BMVC has also been developed as a potential fluorescent marker for cancer cells [25,26]. Whereas BMVC was first developed to detect G4 structures in human telomeres, our recent study shows that BMVC binds the MYC promoter G4 (MycG4, Figure 1b) with higher selectivity and affinity [27]. We have determined the solution structures of BMVC-MycG4 complexes, which show that BMVC binds to the MycG4 via multiple interactions, including stacking external G-tetrads, recognition of the MycG4-flanking bases, and conformational adjustment of the BMVC molecule. Moreover, our results show BMVC represses MYC expression in a human breast cancer cell line. However, the binding selectivity of BMVC to potential G4s formed in the human chromatin has not been broadly examined.

Microarray glass slides with hundred thousands of DNA sequences are a fast, straightforward, and high-throughput platform that has been employed to screen, profile, and quantify ligand and protein interactions with DNA and RNA molecules [28,29,30]. We have very recently designed custom DNA microarrays that can assess the binding selectivities of proteins, small molecules, and antibodies across over 15,000 potential G4 structures [31].

Herein, we report a binding-selectivity analysis of BMVC to the MycG4 and other G4 structures using custom G4 microarrays and competition experiments between Cy5-fluorophore (λ_ex,max_ = 647, λ_em,max_ = 665) labeled small molecule pyridostatin [32] (Cy5-PDS) and unlabeled BMVC. Our results show that BMVC differentially binds to various G4 structures and has a different G4 selectivity profile from Cy5-PDS. BMVC shows preferential binding to the MycG4 among the known G4 structures. Moreover, the microarray data reveals the sequence selectivity of BMVC to the flanking residues of the MycG4, especially at the 3′-end. The large-scale microarray results are confirmed by orthogonal small-scale NMR and fluorescence binding analyses. This is the first large-scale study of a G4-interactive ligand that shows a high-throughput evaluation of G4-binding selectivity and sequence specificity with unbiased selection of G4 sequences. It demonstrates the potential of custom DNA microarrays in the development of drugs targeting DNA or RNA structures.

## 2. Results

### 2.1. BMVC Binds G4 Sequences Differently from PDS

We have designed custom G4 microarrays that contain a total of 19,249 G4 DNA sequences [31]. The G4 microarrays were created by covalently attaching thousands of unique G4-forming DNA 60-mers to a glass surface. Pyridostatin (PDS) is a known G4-interactive compound. Measured by the fluorescence intensity of Cy5-PDS bound to each sequence in potassium-containing solution, Cy5-PDS was shown to preferentially bind G4-forming sequences on the G4 microarrays [31]. To test the binding selectivity of BMVC, we performed competition experiments using custom G4 microarrays (Figure 1 and Figure S1). The addition of potassium-containing solution to G4-forming oligonucleotides induced G4 formation. Subsequently, the microarrays were incubated with 1 µM Cy5-PDS in the absence or presence of 1 µM, 3 µM, or 10 µM of the unlabeled BMVC molecule. After washing to remove the unbound Cy5-PDS and BMVC, the fluorescence intensities of Cy5-PDS bound to DNA oligonucleotides were detected using a fluorescence scanner. The binding selectivity of BMVC to different G4 structures was assessed by measuring the relative fluorescence intensity reduction of Cy5-PDS as BMVC concentration increased.

The fluorescence intensities of 1 µM Cy5-PDS in the presence of various concentrations of unlabeled BMVC were plotted against the fluorescence intensities in the absence of BMVC (Figure 1c). The competition experiment of 1 µM Cy5-PDS with 1 µM of unlabeled PDS was performed as the positive control (Figure S2). For a compound that competitively binds all sequences with the same affinity as Cy5-PDS, the competition experiments of 1 µM Cy5-PDS with various concentrations of the unlabeled compound will follow the predicted linear relationships (Figure 1d). Furthermore, fluorescence intensities of Cy5-PDS bound to various G4 sequences will uniformly decrease in a dose-dependent manner, as presented by decreased slopes (Figure 1d). In the competition experiment, the unlabeled BMVC could compete with the Cy5-PDS binding to G4 sequences in a dose-dependent manner (Figure 1c). However, the binding profile of BMVC was different from unlabeled PDS. Selectivity can be better assessed at equimolar concentrations of unlabeled ligand and Cy5-PDS (both 1 µM) (Figure 1c, left and Figure S2). BMVC displays a more pronounced binding selectivity to different G4 sequences, as shown by a larger deviation from linear relationships, particularly with the stable-G4 forming sequences (at higher fluorescence intensities, Figure 1c, left). Unlabeled PDS appears to bind less selectively to the G4 sequences than BMVC, as shown by the stronger competition at the weaker Cy5-PDS-bound sequences (non-G4 sequences) (at lower fluorescence intensities, Figure S2).

### 2.2. BMVC Shows Different Binding Selectivity to Various G4 Structures as Compared to PDS

**Figure 2:**
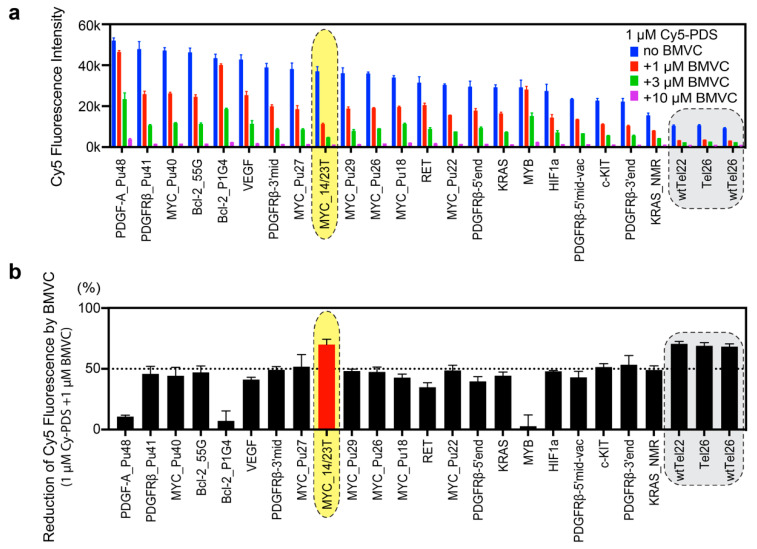
The binding preference of BMVC to known G4 structures. Among the known G4 structures, BMVC prefers to bind to MYC_14/23T, 5′-TGAGGGTGGGTAGGGTGGGTAA-3′ (highlighted by yellow shade). Telomeric sequences are known to form nonparallel structures [42,43,44,45,46] and are poorly bound by Cy5-PDS (shaded in gray). (**a**) The competition microarray experiments showing dose-dependent inhibitory effects of BMVC on the binding of Cy5-PDS to various known G4 structures. The G4 sequences are shown in Table 1. *n* = 2 to 20 independent measurements. Error bars represent mean ± SD. (**b**) BMVC has different inhibitory effects on the binding of Cy5-PDS to the known G4 structures at the equal molar concentration (1 µM). *n* = 2 to 20 independent measurements. Error bars represent mean ± SD.

To determine the G4-binding selectivity of BMVC, we examined the BMVC binding to known G4 structures, including 7 well-studied MYC promoter G4 sequences, 15 other oncogene promoter G4 sequences, and 3 human telomeric G4 sequences (Table 1). BMVC competes with the binding of Cy5-PDS to most G4 sequences in a dose-dependent manner as indicated by reduced fluorescence intensities (Figure 2a).

Comparison of the inhibitory effects for the known G4 structures revealed differential G4 binding selectivity of BMVC vs. Cy5-PDS (Figure 2b). We first ranked G4 sequences based on the fluorescence intensity of bound Cy5-PDS. As illustrated in Figure 2a (blue bars), Cy5-PDS prefers long and highly G-rich sequences, such as PDGF-A_Pu48, PDGFRb_Pu41, and MYC_Pu40. In addition, it also binds well to parallel G4s, such as Bcl-2_55G, Bcl-2_P1G4, VEFG, and various MYC G4s. For most G4s, the fluorescence intensity of 1 µM Cy5-PDS was reduced by 50% upon equimolar addition of BMVC (Figure 2b), suggesting a similar binding affinity of BMVC and Cy5-PDS to these G4s. However, the binding of BMVC was much weaker for the PDGF-A_Pu48 and MYB sequences than the binding of Cy5-PDS (Figure 2b). Both PDGF-A_Pu48 and MYB sequences have no-5′-flanking, while MYB forms a tetrad-heptad structure [54], whereas the optimal binding of BMVC requires a flanking base at both the 5′-end and 3′-end, as shown by NMR solution structural study of the BMVC-MycG4 complex [27] (Figure S3).

Cy5-PDS binds strikingly poor to nonparallel G4s, such as human telomeric G4s, which show less than 25% fluorescence intensity as compared to parallel-stranded G4s (Figure 2b). BMVC significantly inhibited the binding of Cy5-PDS to human telomeric G4s (Figure 2b), indicating a stronger binding of BMVC to the human telomeric G4s as compared to PDS. However, fluorescence measurements showed that BMVC binds parallel G4s, such as MYC and VEGF G4s, much stronger than the human telomeric G4s (Figure S4). Therefore, the microarray competition result indicates that Cy5-PDS binds the human telomeric G4s even weaker than BMVC.

### 2.3. BMVC Preferentially Binds to MYC_14/23T among the Known G4 Structures

In general, Cy5-PDS and BMVC both strongly bind to parallel G4s (Figure 2b). Intriguingly, among all parallel G4s, BMVC induced largest reduction of the Cy5-PDS binding to the MYC_14/23T G4. It is important to note that Cy5-PDS also binds the MYC_14/23T G4 sequence very well (Figure 2a), therefore the strongest competition effect demonstrates that BMVC selectively recognizes the MYC_14/23T G4. The MYC promoter G4 is the best-studied promoter G-quadruplex structure and a prototype of parallel G4s [8]. Notably, MYC_14/23T and MYC_Pu22 form the same parallel G4 (Figure 1b) except for the 3′-end flanking residue, which is a T in MYC_14/23T and a G in MYC_Pu22 [35]. The strikingly stronger binding of BMVC to MYC_14/23T than MYC_Pu22 (Figure 2b) indicates that BMVC selectively recognizes the 3′-flanking T of MYC_14/23T G4.

### 2.4. BMVC Selectively Recognizes the Flanking Sequences of Parallel G4s, Especially the 3′-Flanking T

**Figure 3:**
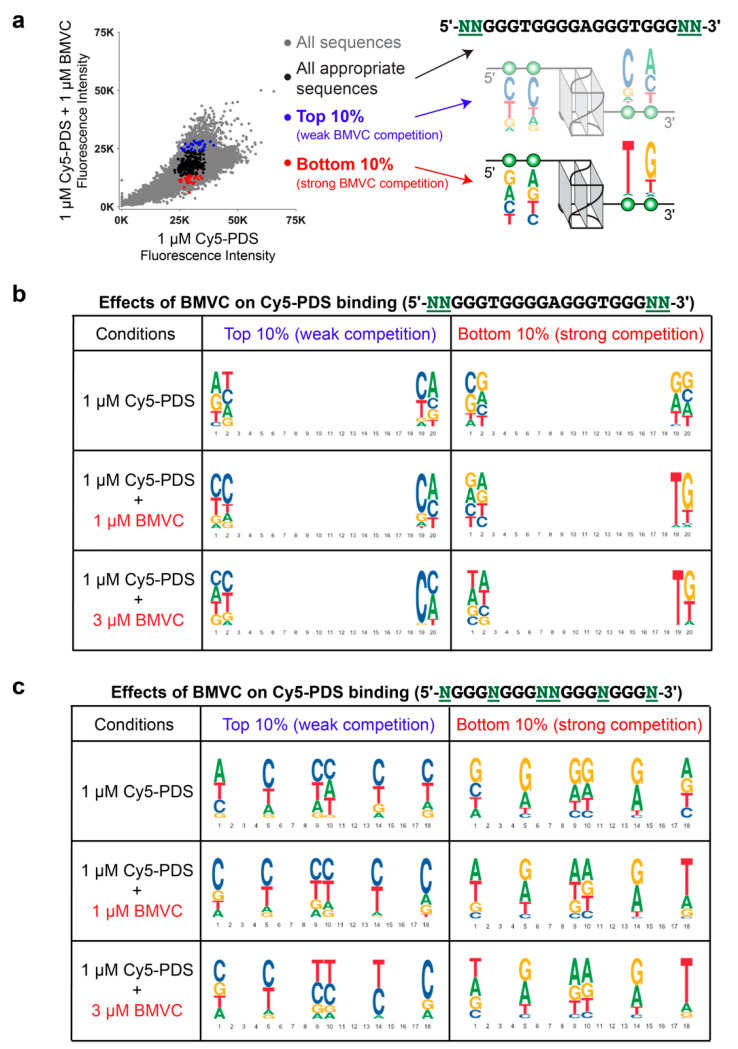
The binding selectivity of BMVC for the flanking sequences of MYC G4. (**a**) An illustration of binding logo analysis for Cy5-PDS signal with the competition of 1 µM BMVC on MYC G4-derived sequences 5′-NNGGGTGGGGAGGGTGGGNN-3′ (256 total sequences). (**b**) The inhibitory effects of BMVC on the binding of Cy5-PDS to MYC G4-derived sequences with variant 5′- and 3′- flanking segments (5′-NNGGGTGGGGAGGGTGGGNN-3′). (**c**) The inhibitory effects of BMVC on the binding of Cy5-PDS to MYC G4-derived sequences 5′-NGGGNGGGNNGGGNGGGN-3′, (4096 total sequences), which include all possible loop and flanking variants.

To examine the preference of BMVC for specific flanking sequences, we analyzed the binding of BMVC to MYC G4-derived sequence variants of the two flanking bases at both ends (5′-NNGGGTGGGGAGGGTGGGNN-3′) using the competition microarray experiments. The differential reduction of Cy5-PDS binding to variants in the flanking sequences induced by BMVC addition reveals the binding selectivity for specific MYC G4 flanking sequences (Figure 3a). In the absence of BMVC, Cy5-PDS exhibits a slight preference for the 3′-flanking C and T, as shown by the most-bound (top 10%) and least-bound (bottom 10%) flanking variants (Figure 3b, top panel). The addition of BMVC significantly altered the most and least Cy5-PDS-bound flanking variants, with a clearly stronger selectivity at the 3′-end than at the 5′-end (Figure 3b, middle and bottom panels). The most and least Cy5-PDS-bound flanking variants in the presence of equimolar BMVC reveal the binding selectivity of BMVC. Particularly, thymine became markedly less enriched in the top 10% Cy5-PDS most-bound 3′-flanking variants but significantly enriched in the bottom 10% Cy5-PDS least-bound variants, indicating that BMVC strongly prefers the MYC G4 with the 3′-flanking T. On the other hand, C is the least-favored flanking base for BMVC binding at both the 3′- and 5′- ends, as shown by the greater enrichment in the top 10% Cy5-PDS most-bound flanking variants.

We further analyzed the effects of BMVC on the Cy5-PDS-binding to MYC G4 loop and single flanking-base sequence variants (5′-NGGGNGGGNNGGGNGGGN-3′), which include all possible loop and flanking variants (Figure 3c). Consistent with the two-base-flanking variants, the results showed BMVC strongly preferred the 3′-end flanking T but disfavored the flanking C at both ends. In contrast, Cy5-PDS preferred C for all three loops and the 3′-end flanking. It is noted that the MYC G4 single flanking-base variants all contain additional 3′-flanking bases for linking the G4 oligos to the microarray plates.

The sequence selectivity shown by the flanking variants explains the markedly weaker binding of BMVC to Bcl-2_P1G4 (Figure 2). Bcl2_55G and Bcl-2_P1G4 both form parallel G4s with a long central loop (13-nt long in Bcl2_55G and 12-nt long in Bcl-2_P1G4) but different flanking sequences [47,48]. However, whereas BMVC showed good binding to Bcl2_55G similar to other parallel G4s, the binding to Bcl-2_P1G4 was markedly weaker (Figure 2a,b). Bcl-2_P1G4 has a flanking C at both the 5′- and 3′- ends and only contains a short 1-nt flanking at the 5′-end, suggesting that BMVC disfavors the flanking C and short flanking.

### 2.5. NMR Binding Experiments Confirm the Binding Selectivity of BMVC to G4 Structures and Flanking Sequences

The binding selectivity of BMVC to G4 structures and flanking sequences was confirmed by NMR titration experiments of BMVC to different G4 sequences, including parallel-stranded MYC_14/23T G4 and its 5′- and 3′-flanking variants, VEGF and MYC1234 G4s, basket-type human telomeric G4 (wtTel22 in Na^+^), and hybrid type human telomeric G4 (Tel26 in K+) (Figures S5 and S6). BMVC binds best to the MYC_14/23T G4, as indicated by well-resolved imino proton peaks for BMVC complexes (Figure S5a). Our previous NMR solution structural study shows that BMVC binds at both ends of the MYC_14/23T G4 to form a 2:1 complex [27] (Figure S3). Mutations at the 5′-flanking sequence do not affect the binding of BMVC at the 5′-end (Figure S6c–e). In contrast, the 3′-end binding of BMVC is sensitive to the mutations at the 3′-flanking sequence, with a clear preference for the 3′-flanking T (Figure S6b). In addition, BMVC prefers at least two flanking bases for a specific binding (Figure S6). These results are in good agreement with the DNA microarray data (Figure 3).

While BMVC binds the MYC_14/23T G4 with the highest affinity (Figure S4), BMVC can bind well to other parallel G4s, such as MYC1234 and VEGF G4 (Figure S5b,d). Additionally, BMVC favors the 5′-flanking A of parallel G4s, as indicated in the NMR titration data of the VEGF G4 flanking variants (Figure S5c,d). However, BMVC did not show specific binding to the basket-type or hybrid-type human telomeric G4s (Figure S5e,f). These results are consistent with the G4 microarray data.

## 3. Conclusions

We have established a high-throughput, large-scale custom G4 DNA microarray to assess the binding selectivities of proteins and small molecules across ~20,000 potential G4 structures simultaneously. Competition binding experiments of the Cy5 labeled PDS and the unlabeled G4-interactive small molecule BMVC demonstrate that the custom G4 microarray platform can assess the binding selectivity of BMVC to various G4 structures and flanking sequences, as well as differential G4 binding selectivity between BMVC and PDS. Our results reveal that BMVC selectively binds parallel G4s, in particular the MYC_14/23T G4. Moreover, the G4 microarray data shows BMVC selectively recognizes the flanking sequences of parallel G4s, especially the 3′-flanking T. Importantly, the binding and sequence selectivity revealed by the large-scale DNA microarray data is in good agreement with the individual binding data by NMR and fluorescence. Our study demonstrates that the G4 DNA microarray provides a high-throughput and unbiased platform to assess the binding selectivity of G4-targeting molecules on a large scale and can help understand the properties that govern molecular recognition.

## 4. Materials and Methods

### 4.1. Custom G4 DNA Microarray Design

We designed a custom microarray that contains four identical sectors that contain ~177,440 ssDNA 60-mers to examine G4 binding selectivity (NCBI GEO Platform GPL28372). The microarray contains different sets of G4 variants designed to examine several sequence parameters that affect G4 formation and binding selectivity such as loop length, loop sequence, flanking tail sequence, and single nucleotide variants of known G4s [31]. Briefly, the array includes a set of sequences from human telomeres and oncogene promoters known to form G4s with various topologies as positive controls (Table 1) as well as a set of 295 additional G4-forming sequences from the literature [57]. Loop and flanking tail sequences were varied using A, T, G, and C polynucleotide stretches and a subset of combinations, described in [31]. For the flanking variants, we generated 256 versions of the major MYC G4 with all possible dinucleotide flanking sequences (5′-NNGGGTGGGGAGGGTGGGNN-3′). For the loop sequence variants, we generated 4,096 sequences of the form 5′-NGGGNGGGNNGGGNGGGN-3′. Negative controls include 19 oncogene G4s in which all G-tracts are replaced with either A, T, or C, reverse complements of G4 sequences, as well as a set of 86 published non-G4 sequences [57].

### 4.2. DNA Microarray Binding Experiments

DNA microarray experiments were performed and analyzed as described previously [31]. Microarrays were preincubated with a pH 7.4 phosphate buffer solution with 100 mM potassium for 1 h at room temperature to induce G4 formation. Arrays then were blocked with 4% nonfat dry-milk in a potassium phosphate buffer before incubation with small molecules (Cy5-PDS, Cy5-PDS+BMVC, or Cy5-PDS+PDS) for 1 h at room temperature.

### 4.3. Data Processing and Analysis

Molecule-bound microarrays were scanned with an Agilent G5761A SureScan Dx Microarray Scanner System to detect Cy5 signal at two laser settings (30 and 100 PMT). Spot intensities from microarray images were extracted using Agilent Feature Extraction Software and are reported as raw fluorescence intensities. All binding assays were performed twice with high agreement between replicates (R > 0.8). Microarrays with the fewest number of saturated spots were used for further analysis. Median intensity was then computed for probes containing identical sequence on each microarray. Sequence logos were generated from a position frequency matrix generated from selected sequences using ggseqlogo [58].

### 4.4. NMR Spectroscopy Experiments

G4 DNA oligonucleotides were synthesized using β-cyanoethylphosphoramidite solid-phase chemistry (Applied Biosystem Expedite 8909), as described previously [36]. NMR experiments were performed on a Bruker AV-III-500-HD equipped with a BBFO Z-gradient cryoprobe. DNA samples were heated to 95 °C for 5 min, then cooled slowly for G4 formation. For the 1D 1H NMR experiments, samples contained 100–250 μM DNA in an appropriate buffer solution with 10% D2O for the lock. The titrations were performed by adding increasing amounts of the compounds to the DNA samples in solution.

## Figures and Tables

**Table 1:** G4 sequences analyzed in Figure 2.

Name	G4 Sequence (5′→3′)
MYC_Pu40 [9]	TTATGGGGAGGGTGGGGAGGGTGGGGAAGGTGGGGAGGAG
MYC_Pu29 [9]	TTGGGGAGGGTGGGGAGGGTGGGGAAGGT
MYC_Pu27 [9]	TGGGGAGGGTGGGGAGGGTGGGGAAGG
MYC_Pu26 [33,34]	TTGGGGAGGGTGGGGAGGGTGGGGAA
MYC_Pu22 [35,36]	TGAGGGTGGGGAGGGTGGGGAA
MYC_14/23T [35,36]	TGAGGGTGGGTAGGGTGGGTAA
MYC_Pu18 [37]	AGGGTGGGGAGGGTGGGG
PDGFRβ_Pu41 [38]	GCTGGGAGAAGGGGGGGCGGCGGGGCAGGGAGGGTGGACGC
PDGFRβ-5′end [38]	TTGGGAGAAGGGGGGGCGGCGGGGCA
PDGFRβ-5′mid-vac [39]	AAGGGAGGGCGGCGGGGCA
PDGFRβ-3′mid [40]	AAGGGGGGGCGGCGGGGCAGGGAGGGT
PDGFRβ-3′end [41]	CGGCGGGGCAGGGAGGGTGGACG
wtTel22 [42]	AGGGTTAGGGTTAGGGTTAGGG
Tel26 [43,44,45]	TTAGGGTTAGGGTTAGGGTTAGGGAAA
wtTel26 [45,46]	TTAGGGTTAGGGTTAGGGTTAGGGTTA
Bcl-2_55G [47]	AGGGGCGGGCGCGGGAGGAAGGGGGCGGGA
Bcl-2_P1G4 [48]	CGGGCGGGAGCGCGGCGGGCGGGCGGGC
PDGF-A_Pu48 [49]	GGAGGCGGGGGGGGGGGGGCGGGGGCGGGGGCGGGGGAGGGGCGCGGC
KRAS [50]	AGGGCGGTGTGGGAAGAGGGAAGAGGGGGAGGCAG
KRAS_NMR [51]	AGGGCGGTGTGGGAATAGGGAA
VEGF [52]	CGGGGCGGGCCGGGGGCGGGGT
RET [53]	GGGTAGGGGCGGGGCGGGGCGGGGGC
MYB [54]	GGAGGAGGAGGTCACGGAGGAGGAGGAGAAGGAGGAGGAGGA
HIF1a [55]	GGGAGGGAGAGGGGGCGGG
c-KIT [56]	AGGGAGGGCGCTGGGAGGAGGG

## Supplementary Materials

The following are available online. Figure S1: Comparison of replicate fluorescence intensity of Cy5-PDS in the absence and presence of 1, 3, and 10 µM of BMVC; Figure S2: Competition microarray experiments of Cy5-PDS with unlabeled PDS; Figure S3: NMR solution structure of the 2:1 complex of BMVC and MYC_14/23T G4 (PDB ID: 6O2L); Figure S4: Apparent dissociation constant (Kd, app) of BMVC binding to various G4s; Figure S5: 1D 1H NMR titration spectra of BMVC with various G4 sequences; Figure S6: 1D 1H NMR titration spectra of BMVC with MYC_14/23T flanking variants.

## Notes

Data (raw probe intensities and processed median intensities) are available at the NCBI GEO database under accession GSE148419.
