# Supplementary material for: Custom G4 Microarrays Reveal Selective G-Quadruplex Recognition of Small Molecule BMVC: A Large-Scale Assessment of Ligand Binding Selectivity
Source: Molecules. 2020 Jul 30;25(15):3465. doi: 10.3390/molecules25153465 (PMC7436161; doi:10.3390/molecules25153465)
Supplement: Supplementary file 1 [file molecules-25-03465-s001.pdf]

Supporting Information

Custom G4 microarrays reveal selective G-quadruplex recognition of small molecule BMVC: a large-scale assessment of ligand binding selectivity

Guanhui Wu[1‡], Desiree Tillo[2‡], Sreejana Ray[2‡], Ta-Chau Chang[3], John S. Schneekloth, Jr.[4], Charles Vinson[2], and Danzhou Yang[1,5,6*]

Table of Contents

Figure S1. Comparison of replicate fluorescence intensity of Cy5-PDS in the absence and presence of 1, 3, and 10 μM of BMVC…………………………………………………………...3

Figure S2. Competition microarray experiments of Cy5-PDS with unlabeled PDS... ……..…...4

Figure S3. NMR solution structure of the 2:1 complex of BMVC and MYC_14/23T G4 (PDB ID: 6O2L) ..……………..………………………………………………………………………5

Figure S4. Apparent dissociation constant ($K_{d, app}$) of BMVC binding to various G4s………...6

Figure S5. 1D ^{1}H NMR titration spectra of BMVC with various G4 sequences…………………...7

Figure S6. 1D ^{1}H NMR titration spectra of BMVC with MYC_14/23T flanking variants……...8

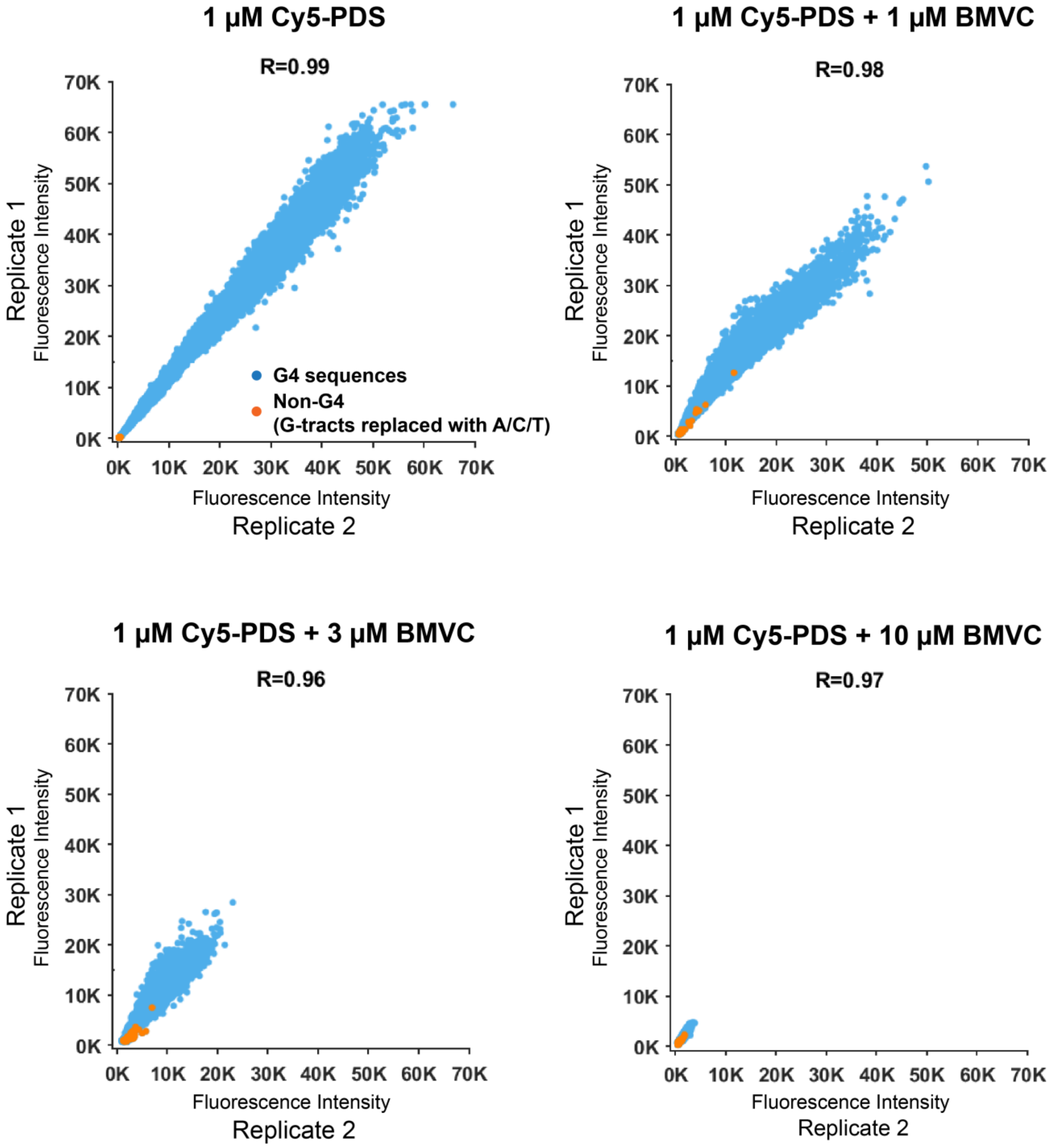

Figure S1. Comparison of replicate fluorescence intensity of Cy5-PDS in the absence and presence of 1, 3, and 10 μM of BMVC. R stands for the Pearson correlation coefficient, which measures the correlation between the *X* and *Y* values of each plot.

a

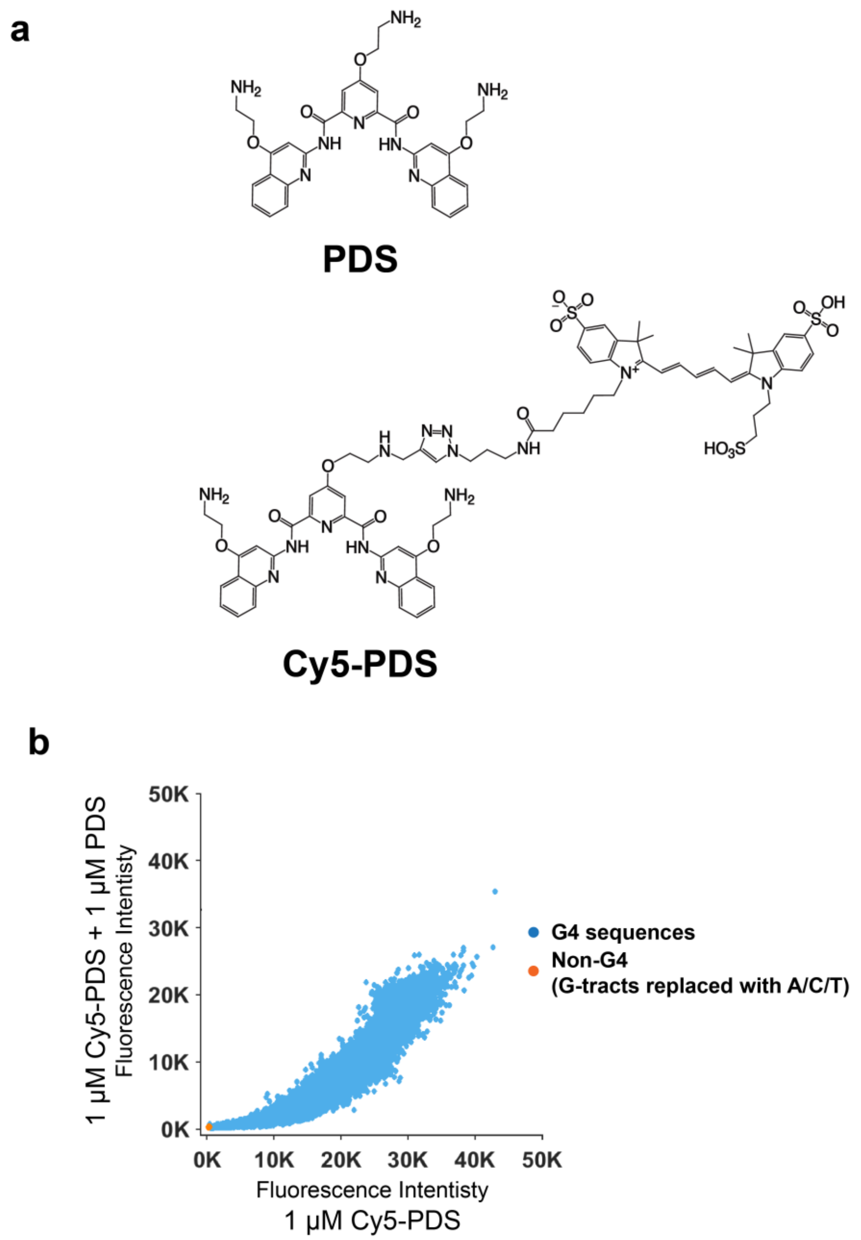

Figure S2. (**a**) Chemical structures of PDS and Cy5-PDS. (**b**) The competition microarray experiments of 1 µM Cy5-PDS with 1 µM unlabeled PDS. The scatterplot showing the fluorescence of 1 µM Cy5-PDS in the presence of 1 µM unlabeled PDS vs. the fluorescence of 1 µM Cy5-PDS in the absence of PDS.

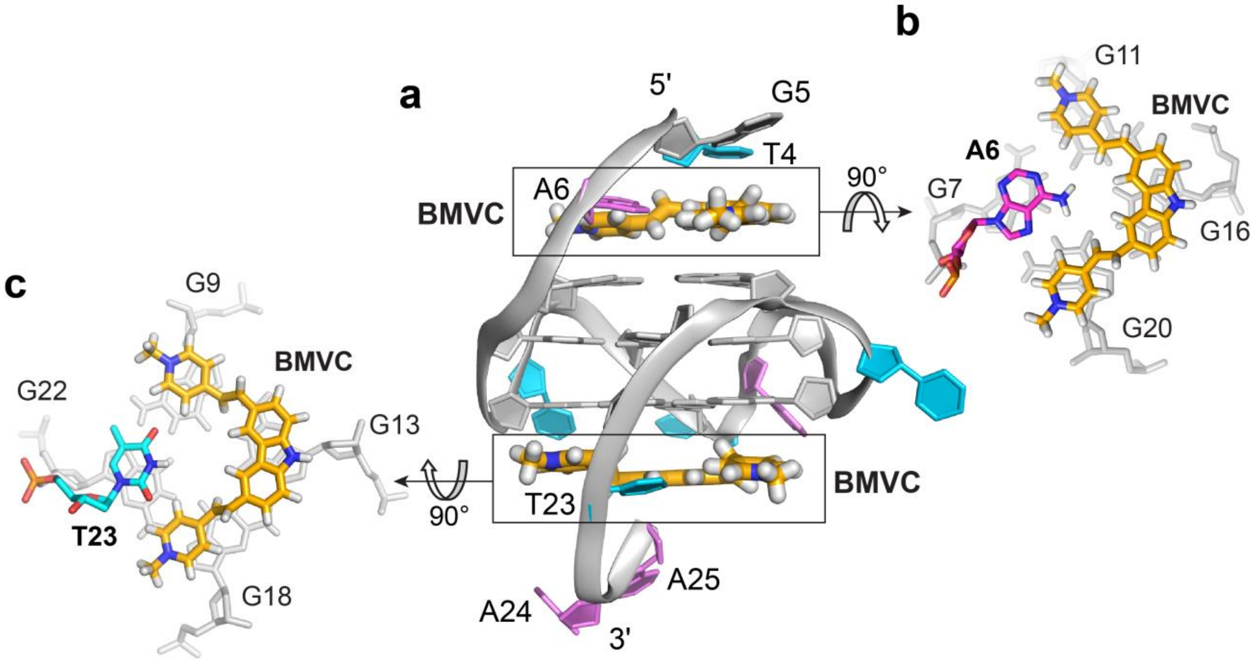

Figure S3. The NMR solution structure of a 2:1 complex of BMVC and MYC_14/23T G4 (PDB ID: 6O2L). (**a**) BMVC (stick model) binds at both the 5'-end and 3'-end of MYC_14/23T G4 (cartoon representation). (**b-c**) For the specific binding, BMVC recruits a flanking base to form a ligand-base pair at both of the 5'-end binding site **(b)** and the 3'-end binding site **(c)**. BMVC = orange, Thymine = cyan, Adenine = magenta, Guanine = gray.

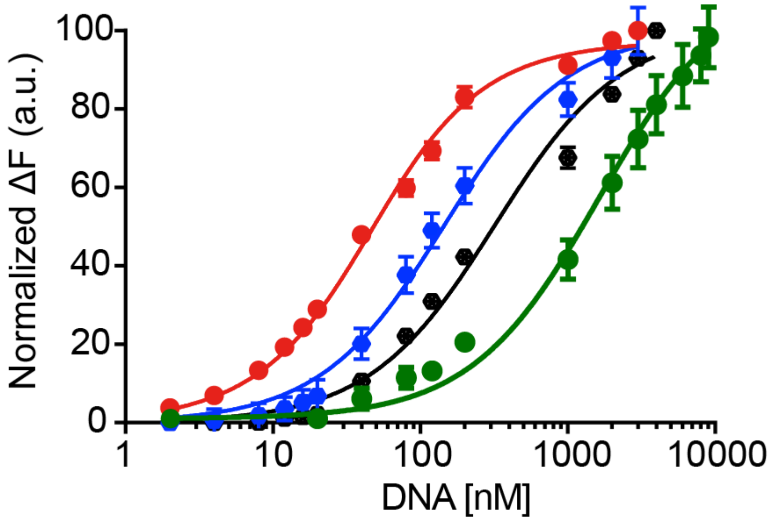

DNA	$K_{d,app}$ (nM)
MYC_14/23T	36 ± 2
VEGF	134 ± 12
wtTel22 (K^+)	311 ± 29
wtTel22 (Na^+)	1614 ± 208

Figure S4. Apparent dissociation constant ($K_{d,\ app}$) of BMVC binding to various G4 structures determined by BMVC fluorescence. Conditions: 20 nM BMVC, 25 °C, pH 7, 100 mM K^+ (100 mM Na^+ for wtTel22).

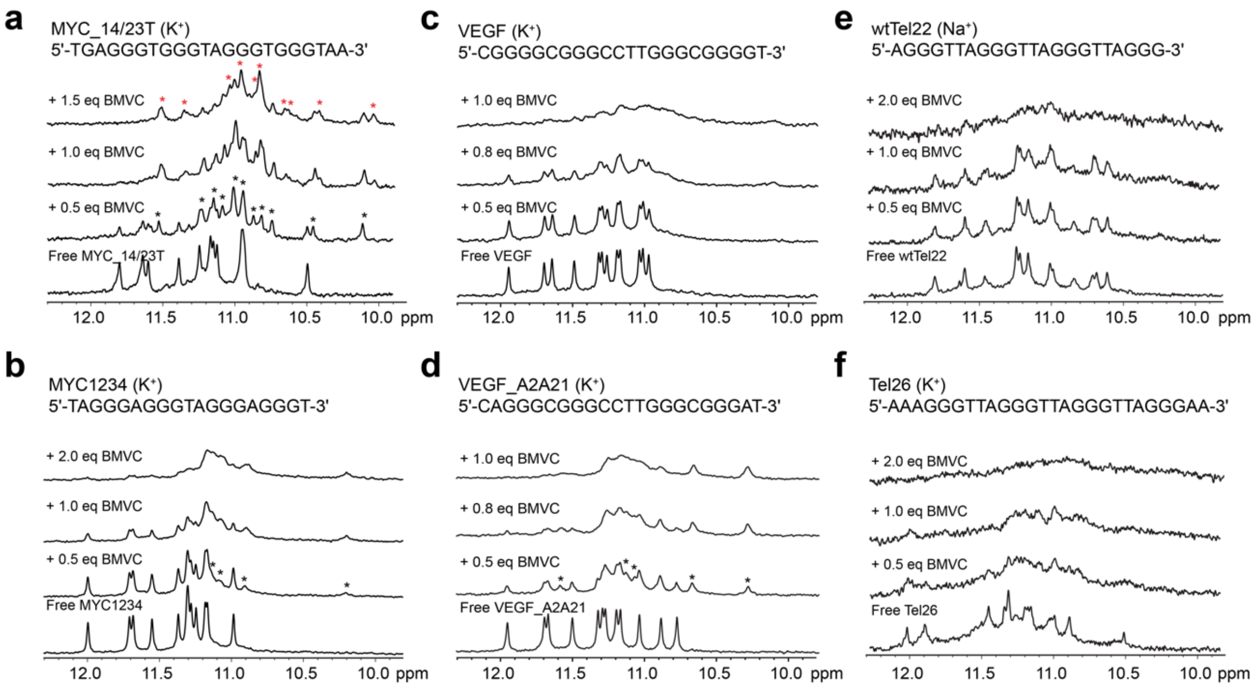

Figure S5. Imino proton regions of the 1D 1H NMR titration spectra of BMVC with various G4 sequences. (**a**) MYC_14/23T, the major parallel G4 formed from the MYC promoter NHE III_1. (**b**) MYC1234, a parallel G4 formed from the 5'-end runs of guanines of the MYC promoter NHE III_1. (**c**) VEGF, a parallel G4 formed in the VEGF promoter. (**d**) VEGF_A2A21, the major parallel G4 formed from the VEGF promoter. (**e**) wtTel22 human telomeric sequence forms a basket-type G4 in Na^+ solution. (**f**) Tel26, the hybrid-1 human telomeric G4. Imino protons arising from the 1:1 or 2:1 complex formation are marked with asterisks in black or red, respectively. All spectra were collected at 25 °C, pH=7.

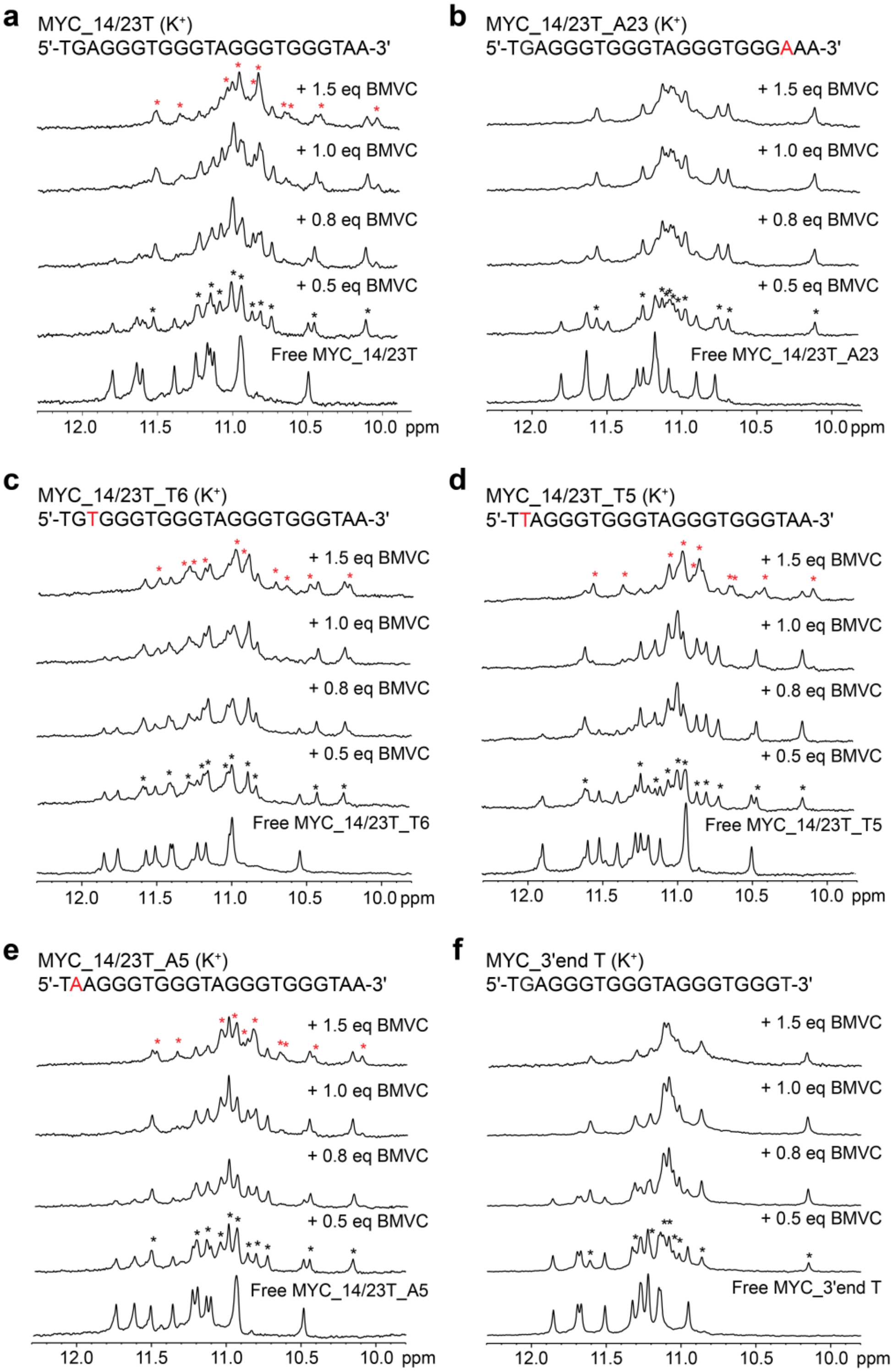

Figure S6. Imino proton regions of the 1D ^{1}H NMR titration spectra of BMVC with MYC_1423T G4 (**a**), its 3'-end modified (**b, f)** and 5'-end modified (**c-e**) sequences. Imino protons arising from the 1:1 or 2:1 complex formation are marked with asterisks in black or red, respectively. All spectra were collected in 95 mM K^+, pH=7 solution, at 25 °C.
